# Assessing Head Acceleration Events in Female Community Rugby Union Players: A Cohort Study Using Instrumented Mouthguards

**DOI:** 10.1007/s40279-024-02111-3

**Published:** 2024-09-05

**Authors:** Melanie D. Bussey, Danielle Salmon, Bridie Nanai, Janelle Romanchuk, Raul M. Gomez, Darryl Tong, Gisela Sole, Ross Tucker, Éanna Falvey

**Affiliations:** 1 School of Physical Education, Sports and Exercise Sciences, University of Otago, Dunedin, New Zealand; 2 New Zealand Rugby, Wellington, New Zealand; 3 Institute of Sport and Exercise Medicine, University of Stellenbosch, Stellenbosch, South Africa; 4 World Rugby, Dublin, Ireland; 5 School of Medicine & Health, University College Cork, Cork, Ireland; 6 Department of Oral Diagnostic and Surgical Science, University of Otago, Dunedin, New Zealand; 7 School of Physiotherapy, University of Otago, Dunedin, New Zealand

## Abstract

**Background:**

The rapid growth of women's rugby union has underscored the need for female-specific player welfare protocols, particularly regarding the risk of head injuries. Instrumented mouthguards (iMGs) play a vital role in gathering comprehensive data on head acceleration events (HAEs), including their frequency, magnitude, and spatial distribution during games and training. By doing so, iMGs offer valuable context for circumstances in women's matches that may increase player risk.

**Objectives:**

The study aimed to contextualize HAEs in female community rugby players using instrumented mouthguards and video review.

**Methods:**

This prospective, observational cohort study involved 332 female rugby players across 38 matches and 80 training sessions during the 2021/2022 seasons. Players were representative of four playing grades: U13 (*N* = 9), U15 (*N* = 111), U19 (*N* = 95) and Premier women (*N* = 115). HAEs were recorded using boil-and-bite iMGs, with a single-axis recording threshold of 5 g. The incidence and prevalence of HAEs was expressed by grade, years of experience, playing positions, and session types (match or training). The effect of playing grade and previous playing experience on HAE propensity during tackles and rucks was also examined.

**Results:**

Throughout the study, 9151 iMG events over 5 g were recorded, with 80% verified for analysis. Overall, the incidence rate (IR) was highest for HAEs between 10 and 29 g, 12–18 times higher than the IR for > 30-g events. Premier grade players had the highest weekly HAE load (26.2 per player per week) and the highest prevalence of players (49%) exposed to events over 30 g. An inverse relationship was found between years of rugby experience and peak angular acceleration (PAA) in U13–U19 players (*p* = 0.002, 95% CI [47,177 rads/s^2^]), showing that more experienced school-age players had lower rotational acceleration during HAEs. However, propensity for HAEs in tackle events was highest in Premier players with > 9 years of experience compared with U13–U19 grade players with similar years of experience (RR = 1.21, 95% CI 1.06–1.37; *p* = 0.004). Ball carries consistently resulted in the highest propensity of events over 30 g, regardless of playing grade or experience.

**Conclusions:**

This research presents unique information regarding head accelerations that occur during women’s community rugby matches and practices. The results have significant implications for recognising populations that are at the highest risk of experiencing high cumulative and acute head accelerations. The findings may assist in managing training loads and instructing skill execution in high-risk activities, particularly for younger players who are new to the sport.

Consideration of playing grade, experience, and contact phases is crucial for understanding head acceleration exposure and injury risk in female rugby players. These insights can inform injury prevention strategies.

**Supplementary Information:**

The online version contains supplementary material available at 10.1007/s40279-024-02111-3.

## Key Points

**Table:** 

Weekly exposure: Premier women had the highest weekly head acceleration exposure compared with all school-age players. This is likely attributed to the more intense and physical nature of play at the premier level.
Propensity for head acceleration events (HAEs) during contact phases: Within high-experience players, playing grade (Premier) significantly impacts HAE risk in tackle events. Moreover, ball carries pose the greatest risk of > 30-g HAEs across all grades and experience levels.
Years of rugby experience: There was a significant inverse relationship between playing experience and rotational acceleration in U13–U19 players, indicating that improved technique and motor control over time may lower risk in younger players.
Consideration of playing grade, experience, and contact phases is crucial for understanding head acceleration exposure and injury risk in female rugby players. These insights can inform injury prevention strategies.

## Introduction

The women's game is currently the fastest-growing sector of rugby union worldwide, with participation rates rising by 28% annually, and an estimated 2.7 million female participants worldwide [1, 2]. The growth in participation rates has been accompanied by the professionalization of the women’s game with the newly developed WXV and Six Nations competitions [3]. This expansion has been viewed as a progressive development in fostering rugby union as a sport for women as well as men [4]. However, as participation rates climb, so does the scrutiny of specific training and match loads faced by female athletes, highlighting the need for female-specific player welfare protocols [2, 5, 6]. The most pressing concerns revolve around the potential for head injuries sustained during gameplay and the long-term effects of repetitive head accelerations that may not necessarily present clinically at the time [7–9].

A head acceleration event (HAE) in contact sports refers to the sudden increase in the velocity at which a player's head moves during a contact or collision incident [10]. In contact sports like rugby, HAEs are increasingly recognized for their potential link to head injuries, including concussions [11–13]. To this end, instrumented mouthguards (iMGs) have proven indispensable in capturing a spectrum of the HAE context, from frequency and magnitude to the spatial distribution of impact events. This capability allows for accurate quantification of the head acceleration load related to various contact situations during gameplay and training activities [14–16]. Understanding the sporting context of head acceleration thresholds is crucial for gaining insights into how head kinematics might influence clinical outcomes and aid in player welfare strategies.

Contextualising the head acceleration load in community players is critically important to the player welfare strategy because it represents the grass roots of the sport and a player’s first exposure to rugby. For example, McIntosh et al. [17] examined head, face and neck injuries in community rugby and identified that rucks and mauls posed significant risks. This study recommended modifying playing rules at the grassroots level to enhance safety. As a result, changes included stricter enforcement of existing rules to prevent dangerous play, better training for coaches and referees on safe techniques, and the introduction of mandatory safety protocols during rucks and mauls. While research has explored head acceleration exposure in male community rugby players using iMGs [10, 18, 19], limited data exist on female community players [18, 20]. This is particularly important in the women’s space, due to the rapid growth of the sport and the later stage of entry of female players [1]. As participation grows, community-level coaches grapple with managing players with diverse abilities, maturity levels, and experiences. This knowledge gap impedes our understanding of head acceleration exposure in this specific demographic and the formulation of tailored evidence-based mitigation strategies.

The purpose of this study is to investigate and contextualize the incidence, incidence thresholds, and propensity of HAEs in female community rugby players, leveraging iMG technology. The study aims to address the knowledge gap regarding head acceleration exposure in this specific demographic by considering diverse playing experiences and competitive grades, as well as factors such as players' first exposure to rugby. This research will contribute to the knowledge base for evidence-informed mitigation strategies for player welfare in female community rugby.

NOTE: This manuscript focuses on female participants in the Otago Rugby Community Head Impact Detection (ORCHID) study, which is a larger research project that investigates head acceleration events in over 700 community rugby players. The decision to subset the total project by sex was made because of the substantial differences in exposure and playing experience between male and female rugby players. By focusing solely on female participants, the authors aimed to provide a more detailed examination of head acceleration events within this particular subgroup of community rugby players. This approach allows the analysis of the data in the appropriate exposure context, considering the unique characteristics and experiences of female rugby players, with a focus on playing grade and experience.

## Methods

### Participants

This prospective observational cohort study analysed HAEs collected through iMGs in 332 female players across four age grades in 38 matches and 80 trainings over the 2021/2022 seasons. Participants were recruited from local clubs and schools, and represented the spectrum of community rugby where contact was permitted. Informed consent was obtained from all players, legal guardian consent was also obtained for all players under the age of 16 years. A detailed demographic breakdown is provided in Table 1. This study was conducted in accordance with the ethical principles outlined in the Declaration of Helsinki, and ethical approval was granted by the university ethics committee (approval number H21/056).

**Table 1:** Female player demographic and rugby experience variables for each grade for the 2021 and 2022 season

Playing grade	Secondary School or Club	Age (y)			Height (cm)			Weight (kg)			Rugby experience (y)			Position		
		*n*	Mean	SD	*n*	Mean	SD	*n*	Mean	SD	*n*	Mean	SD	*n*	Forward	Backs
U13	Club	9	13.5	0.7	7	163.0	5.9	7	59.6	8.1	9	2.7	1.4	9	4 (44%)	5 (55%)
U15–2021	Secondary	59	14.0	0.9	48	164.3	8.1	49	58.4	9.2	52	1.8	1.5	51	16 (31%)	35 (69%)
U15–2022	Secondary	54	14.3	1.0	54	166.6	8.1	54	65.9	11.5	54	2.8	2.1	52	27 (52%)	25 (48%)
U19	Secondary	95	15.8	1.3	90	165.4	6.4	89	67.4	12.0	93	3.8	2.4	92	48 (52%)	44 (48%)
Premier	Club	115	21.2	3.7	114	168.2	7.2	115	78.1	14.1	114	7.0	5.5	115	59 (51%)	56 (49%)

Several players played in multiple grades; if a player played in two different grades, their data was included in the demographic details for both grades. The following outlines the crossover**: U13**: one player played in U13 and U15–2021; one player played in U13, U15–2021, and U15–2022; four players played in the U13 grade and the U19 grade; **U15:** 19 players played in U15–2021 and U15–2022; three players played in U15–2022 and in U19 grades; **U19**: eight players played in U19 and Premier grades

The duration of matches for secondary school players differed depending upon playing grade and number of players per side, according to the “Game on Provisions” within New Zealand Rugby’s domestic safety laws [21]. The length of a match for school-age players may range from 40 min at minimum (10 a side) to 70 min maximum (15 a side), while the Premier grade played typical 80-min matches. Domestic rules further allow the U13 grade to be played with mixed genders, although only a small number of females participate at this level.

### Study Equipment

As per Bussey et al. (2023), players were provided with a boil-and-bite iMG (Prevent Biometrics®, MN, USA) fitted by a qualified dentist to ensure accurate coupling to the players’ dentition [10]. The iMG contains an infrared proximity sensor, a 3.2 kHz triaxial accelerometer, and gyroscope with measurement ranges of 200 g and 35 rads/s. The proximity sensor assessed the iMG coupling to the players’ dentition (using Prevent Biometrics proprietary calculations) and produced a temporal ‘on-teeth’ log. The iMG was set to trigger when an HAE exceeded a 5-g threshold along any single axis (x, y, z) [22]. The recording window was 50 ms, which encompassed a 10-ms back sample plus a 40-ms post-trigger.

All matches and trainings were recorded in high definition using two mounted cameras (at end-on and side-on angles). For matches, referees wore head-mounted cameras (Hero8, GoPro Inc., USA) that provided a third angle. As per Bussey et al. (2023), camera footage was synchronized and imported into Hudl Sportscode (v 11, Agile Sports Technologies Inc., NB, USA) [10]. The iMG data were converted to XML format and time synchronized to the Sportscode timeline using a clock flash captured in the video. Each player’s unique iMG number was matched to the corresponding player jersey number, allowing the analyst to match the individual player to the iMG event timeline. The video associated with each iMG event was qualitatively analysed to identify the match context (e.g., tackle, carry, ruck, maul, scrum, lineout, running, falling, jumping [23]) and to confirm the HAE mechanism (i.e., direct or indirect contact vs voluntary [10]) for the head acceleration trigger. A tackle was defined as an attempt to halt the progress of an opponent, a carry as an engagement of an opponent whilst carrying the ball [24], and a ruck is formed when at least one player from each team are in contact, on their feet and over the ball which is on the ground [23]. All iMG events over 8 g were video-verified, and events between 5 and 8 g were video-verified if they occurred during a match phase and could be attributed to an HAE mechanism. During the verification process, HAEs that were obscured, where the HAE mechanism could not be confirmed as direct or indirect, were labelled as ‘unclear’, if the player could not be viewed in the video the event was labelled as ‘off-camera’. Unclear events were included in the analysis, but off-camera events were not included as the context for the event could not be verified. As per the Consensus for Head Acceleration Measurement Practices (CHAMP) recommendations, all raw acceleration waveforms associated with the verified or unclear HAEs were inspected for signal quality prior to inclusion in the final dataset [25]. Bussey et al. (2023) provides further examples of the types of signal issues that might lead to the removal of an event [10]. For propensity analysis, we needed to identify the total number of tackles, carries, and rucks that each instrumented player was involved in whilst wearing the iMG. To do this, the iMG timeline was synchronized with independently coded match data in Sportscode, in a similar manner to Tooby et al. [24]. Only contact events that corresponded with an on-teeth period for the instrumented player were used in propensity calculations.

For the trainings, temporal windowing was implemented to align the HAEs with coded training events. This process comprises two steps: iMG and video-time synchronization. In the first step, the proximity sensor data determined the average time of the first ‘on-teeth’ information for each player, with a maximum standard deviation of 3 min. The calculated starting times from this initial step were then added to the relative times from the video files to create a synchronized impact plus the video file. In the second step, a statistical comparison was performed by evaluating the synchronization results using the starting time from step one plus and/or minus 60 s. This determined which value resulted in a higher number of impacts synchronized with the video information. Iterating through this process enabled the selection of starting times for the video file with the greatest number of impacts synchronized with the video information, as well as the highest number of players with proximity data available for each session. The quality of synchronization was visually verified using the Sportscode timeline. Further labelling of HAE events was subsequently completed in Sportscode.

### Data Processing

The raw accelerometer and gyroscope data were downloaded from the Prevent Biometrics server and imported into MATLAB (R2021b, MathWorks Inc., California, USA) for processing. Bespoke scripts were used for data reduction and postprocessing. Angular acceleration was derived using a five-point stencil numerical derivation equation. Data were filtered using a 200 Hz zero phase, 4-pole Butterworth filter [26] before being transformed into the head centre of mass (COM). The location and coordinates for the head COM were categorized as either 5th percentile female (used for athletes who fit in the 5th percentile of the population by height and weight) or 50th percentile female (for everyone else) [27–29].

### Statistical Analysis

All statistical analyses were performed using R (v. 4.3.2; R_Core_Team 2015) and MATLAB. Demographic details, including age, height, weight, and years of rugby experience, are reported using the mean and standard deviation (SD). Incidence was calculated for each player based on the exposure type and time normalized to 60 min (E1) or weekly incidence normalized to min exposure per week (E2). Player exposure time (in minutes) was extracted from the video footage for each instrumented player, whereas the percentage of iMG wear was extracted from the proximity data log and expressed relative to the total session time. The prevalence of HAE > 30-g events was calculated as the percentage of weekly exposed players receiving at least one > 30-g event per session.

$$\left[ {E1} \right] {\text{Player}} {\text{IR}}_{60 } = \frac{{\sum {\text{HAE per threshold band}}}}{{\sum {\text{Total playingminutes}}/60}}$$

$$\left[ {E2} \right] {\text{Player IR}}_{{{\text{week}}}} = \frac{{ \left\lceil {\sum {\text{HAE per threshold band}}} \right\rceil }}{{\left\lfloor {\sum {\text{Total playing or training minutes}}/{\text{week}}} \right\rfloor }}.$$

The analysis of incidence and propensity focused on years of playing experience (low, medium, and high) across two playing grades (school-age vs Premier). This approach enabled playing-grade comparisons *between* adult and school-age players as well as *within*-grade comparisons by experience level. The decision was guided by several key observations of the player demographics (Table 1). Most significantly, by the substantial overlap in years of experience and age between the U13s, U15s, and U19s. Thus, grade alone may not accurately reflect a player's skill, proficiency, or years of playing experience. To better capture the influence of playing experience on the HAE incidence and propensity, the years-of-experience variable was split into three equal groupings (low = 1.5 ± 0.8 y, medium 4.8 ± 0.7 y, high = 9.5 ± 3.3 y) using the cut and quantile functions in R.

Negative binomial regression (glmmTMB package) was used to model the HAE counts between grades (school-age or Premier) and experience levels (high, medium, or low) at or above each threshold (in 10-g or 500-rads/s^2^ increments), with log-time included as an offset. Quantile mixed regression (lqmm and emmeans packages) was employed to model the relationship between years of rugby experience and the peak linear acceleration (PLA) and peak angular acceleration (PAA) across the 4th, 5th and 6th quantiles. Years of experience was included as a covariate, with session type (match or training) and playing position (forward or back) as factors, along with three- and two-way interactions. Separate quantile models were run for each grade (Premier or school-age). Both the negative binomial and quantile models utilize player ID and teams as a crossed random effect. The propensity of HAE was calculated for match sessions as the number of HAEs for each player per contact event divided by the total number of contact events that the player was involved with while wearing the iMG [24]. The relative risk of contact-event HAE was calculated for each experience group [30].

## Results

### Overall Counts

Over the study, 9151 iMG events were captured, 578 events were removed due to errors indicative of sensor malfunction, and 1293 events were identified as false events either via video review or were identified as ‘off-teeth’ or undergoing decoupling/recoupling, as evidenced by proximity logs [31, 32]. This left 7280 HAEs over 5 g for the analysis.

### Weekly Exposure Time and Head Acceleration Event (HAE) Incidence

Over the 2021/2022 seasons, 1990 player exposure hours (1218 player-hours of training, 772 player-hours of matches) were captured. There was a marked difference (~ 90 min) in total rugby exposure between club (U13 and Premier women) and high-school (U15 and U19) players (Table 2). For instance, U13 girls had 56.4% more rugby exposure than U15 girls (163 min − 71 min = 92 min), while weekly exposure in Premier women was 49.2% greater than in U19 women (187 min − 95 min = 92 min) (Table 2).

**Table 2:** Match and training exposure time and percentage of time on teeth for the iMG device for the different grades for female players over the 2021 and 2022 seasons

Player grade	Matches								Trainings					
	Sample size (*n*)	Total matches (*n*)	Matches per player (*n* ± SD)	Duration (min)		Player time (min)		iMG on teeth time (%)	Sample size (*n*)	Total trainings (*n*)	Trainings per player (*n* ± SD)	Duration (min)		iMG on teeth time (%)
				Mean	SD	Mean	SD					Mean	SD	
U13 girls	6	5	2.2 ± 1.3	62	2.6	35.1	13.4	70.5	8	13	3.0 ± 1.4	64.2	22.5	54.1
U15 girls	60^a^	23	3.0 ± 1.4	41.3	12.1	21.2	10.8	51.4	63^b^	23	2.6 ± 2.1	50.2	34.7	49.3
U19 girls	67	14	2.6 ± 1.0	75.0	10.3	40.2	18.0	47.8	66	18	3.0 ± 1.7	55.2	14.4	63.2
Premier women	94	16	4.1 ± 2.1	82.7	10.7	49.8	24.9	51.8	82	32	5.5 ± 3.4	68.8	12.5	43.6

*iMG* instrumented mouthguard, *SD* standard deviation

^a^11 unique to 2021

^b^10 unique to 2021

Exposure differences were accumulated across both training and matches. Club players typically completed two trainings per week, followed by a weekend 15-a-side match. In contrast, the high school players trained only once per week and played one mid-week game, which varied in structure and duration depending on player availability. High-school matches ranged from 14 to 70 min, with the average match contributing 50–60% of total rugby exposure per week. Owing to player availability, the U15s play mostly a 7 s or 10 s game structure, while the U19s played 15 s.

The weekly HAE incidence rates (IR) are presented in Table 3. For all grades during match play, IR 10–29 g was on average 12–18 times higher than IR > 30 g (Table 3). The prevalence of players experiencing > 30-g events increased with grade, 29% in U13 to 49% in the Premier (Table 3). Only the U15s had a higher prevalence of > 30-g HAE in trainings when compared with matches (Table 3). A full breakdown of HAEs per trigger threshold is provided in the supplementary table (see electronic supplementary material [ESM]).

**Table 3:** Weekly incidence rates have been scaled from standard playing hours to average weekly exposure time per player and grade. Prevalence represents the percentage of total players exposed per week who experience a > 30 g event

Player grade	Match exposure (1M)		Training exposure (1T)		Weekly match + training(s)				
	Match incidence rate		Session incidence rate		Typical weekly schedule	Total weekly incidence rate		Match prevalence	Training prevalence
	IR 10–29 g	IR > 30 g	IR 10–29 g	IR > 30 g		IR 10–29 g	IR > 30 g	> 30 g (%)	> 30 g (%)
U13 girls*	8.8	0.6	3.6	0.2	1M + 2T	16.0	1.0	29*	24*
U15 girls	8.2	0.6	6.7	0.8	1M + 1T	14.9	1.4	29	39
U19 girls	9.9	0.8	2.2	0.2	1M + 1T	12.1	1.0	37	11
Premier women	14.6	0.8	4.5	0.9	1M + 2T	23.6	2.6	49	41

Peak linear acceleration (PLA) bins 10–29 included events up to 29.99 g, creating a clear transition between under and over 30 g

Prevalence represents the mean percentage of players in the exposed population who experience an HAE > 30 g

*HAE* head acceleration event, *IR* incidence rate, *M* match, *T* training

*These players were competing in the mixed grade. The IRs and prevalence in the table are calculated for females only. The relative risk for female U13s is 1.18 (95% CI 0.336–4.178; *p* = 0.715) compared with male U13s

### Years of Rugby Experience and HAE Per Player Hour Incidence Rate

**Fig. 1:**
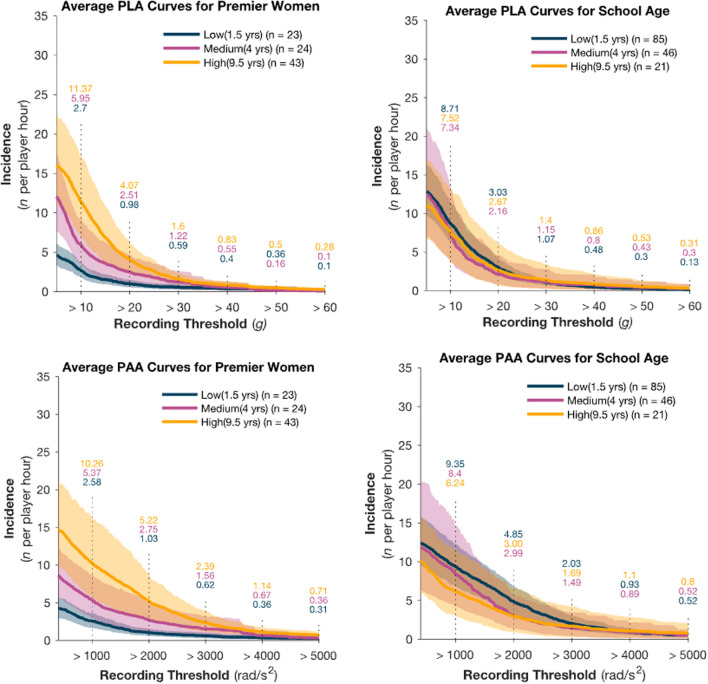
Per-hour incidence rate for head acceleration events (HAEs) by playing experience grouping: low (blue), medium (magenta) and high (yellow). Premier grade figures appear on the left and school-age on the right with PLA (g) incidence rates on top and PAA (rads/s^2^) incidence rates on the bottom. The solid line represents the mean incidence rate and shading represents the 95% confidence interval. *HAE* incident rates per peak acceleration magnitude band are indicated by the numbers above each vertical line. *PAA* peak angular acceleration, *PLA* peak linear acceleration

The total number of HAEs captured during the matches was 4495, while for trainings it was 2785. The normalized HAE IRs per threshold per player hour are presented in Fig. 1. The mean IRs were calculated for each player according to E1, while the 95% confidence interval (95% CI) bands were estimated using a bootstrapping procedure that randomly resampled the dataset 3000 times (Fig. 1). The results of the binomial regression indicated evidence of a significant two-way interaction (grade × experience) for PLA (*X*^2^ = 6.19, *p* < 0.04) and PAA (*X*^2^ = 146.6, *p* < 0.001), the effect of which diminishes with higher thresholds > 30 g PLA and > 4000 rads/s^2^. Generally, the interaction effect indicated the difference between playing grades depends on playing experience. Specifically, School-age players with the lowest years of experience had significantly higher HAE IR than Premier players with the lowest years of experience.

### Years of Rugby Experience and Peak Angular Acceleration (PAA) Magnitude

**Fig. 2:**
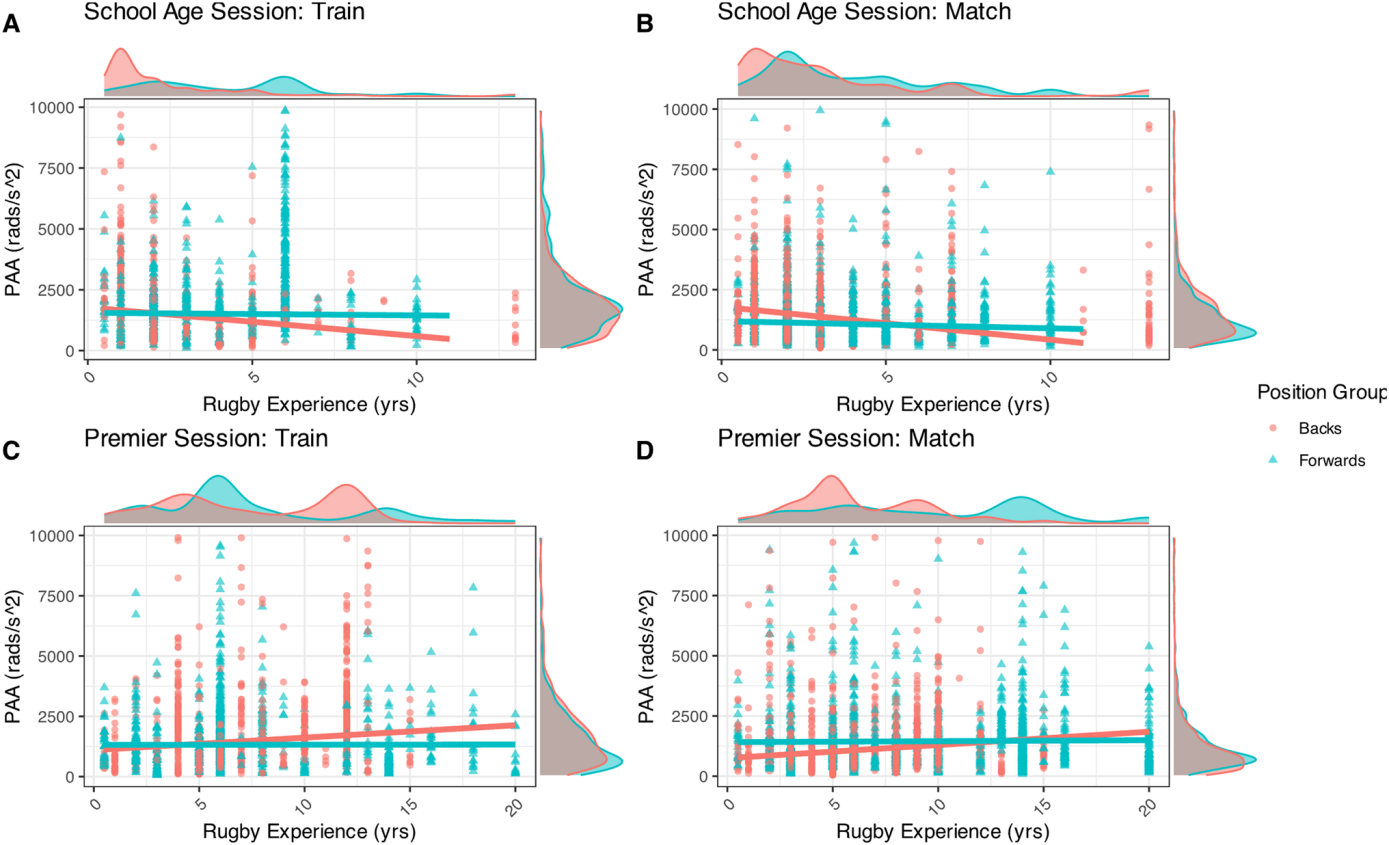
Relationship between rugby experience and peak rotational head acceleration (PAA) by playing position. Each point represents a single HAE, with quantile regression lines illustrating the relationship within each player group. The school-age grade is represented in images A and B while Premier grade is shown in C and D. *HAE* head acceleration event

The findings from the quantile mixed regression model (Fig. 2) demonstrate a noteworthy inverse relationship between years of rugby playing experience and PAA in school-age players. Specifically, the median PAA decreased by 112 rads/s^2^ for each year of experience (*p* = 0.002, 95% CI 177–47), indicating that as school-age players accumulated more rugby experience, they exhibited lower rotational acceleration during HAEs. The results of the school-age training sessions, as depicted in Fig. 2a, indicate an interaction effect between forwards and backs; however, this effect was not statistically significant in the model (Tau = 0.5). In contrast, a significant two-way interaction effect (*p* = 0.016) was observed in Premier women, which was attributable to the significant main effect of position for both matches and trainings. This analysis demonstrates that the association between PAA and years of rugby experience varies by playing position. As shown in Fig. 2, PAA tends to increase with experience among backs, whereas no significant relationship is observed for forwards.

### Propensity of HAE by Contact Event Type

**Fig. 3:**
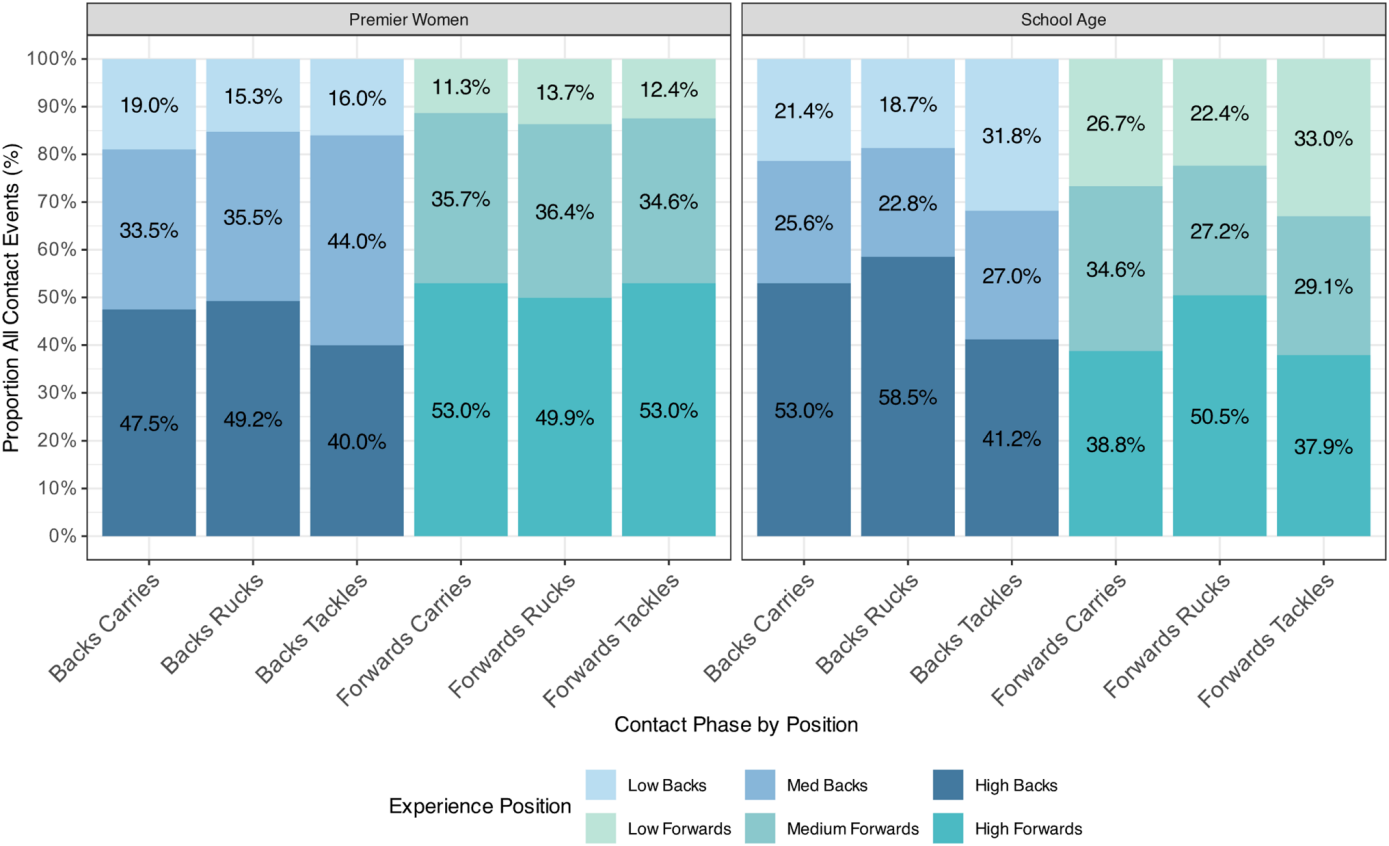
This stacked bar chart visualizes the distribution of contact events, within two player grades: Premier (left bars) and school age (right bars). Each bar is segmented by the frequency of involvement in each contact phase, stratified by experience level (low, medium, high). The percentages displayed within each segment represent the proportion of total contact events attributed to players by playing position

In total, 8518 contact events were identified in the instrumented female cohort. Figure 3 presents the total proportion of contact events ascribed to tackles, carries, and rucks by playing position and level of experience. A common trend was that more experienced players were involved in a higher proportion of carriers, rucks, and tackles. In Premier women, the proportion of events increases consistently with experience and closely reflects the proportion of the population; thus, the most experienced players appear in ~ 50% of contact events, whereas the least experienced players account for < 20% of contact events. In the school-age group, the smallest proportion of players (high experience) accounted for the largest proportion of contact events.

#### HAE Propensity: Effect of Grade Within Experience Level

**Fig. 4:**
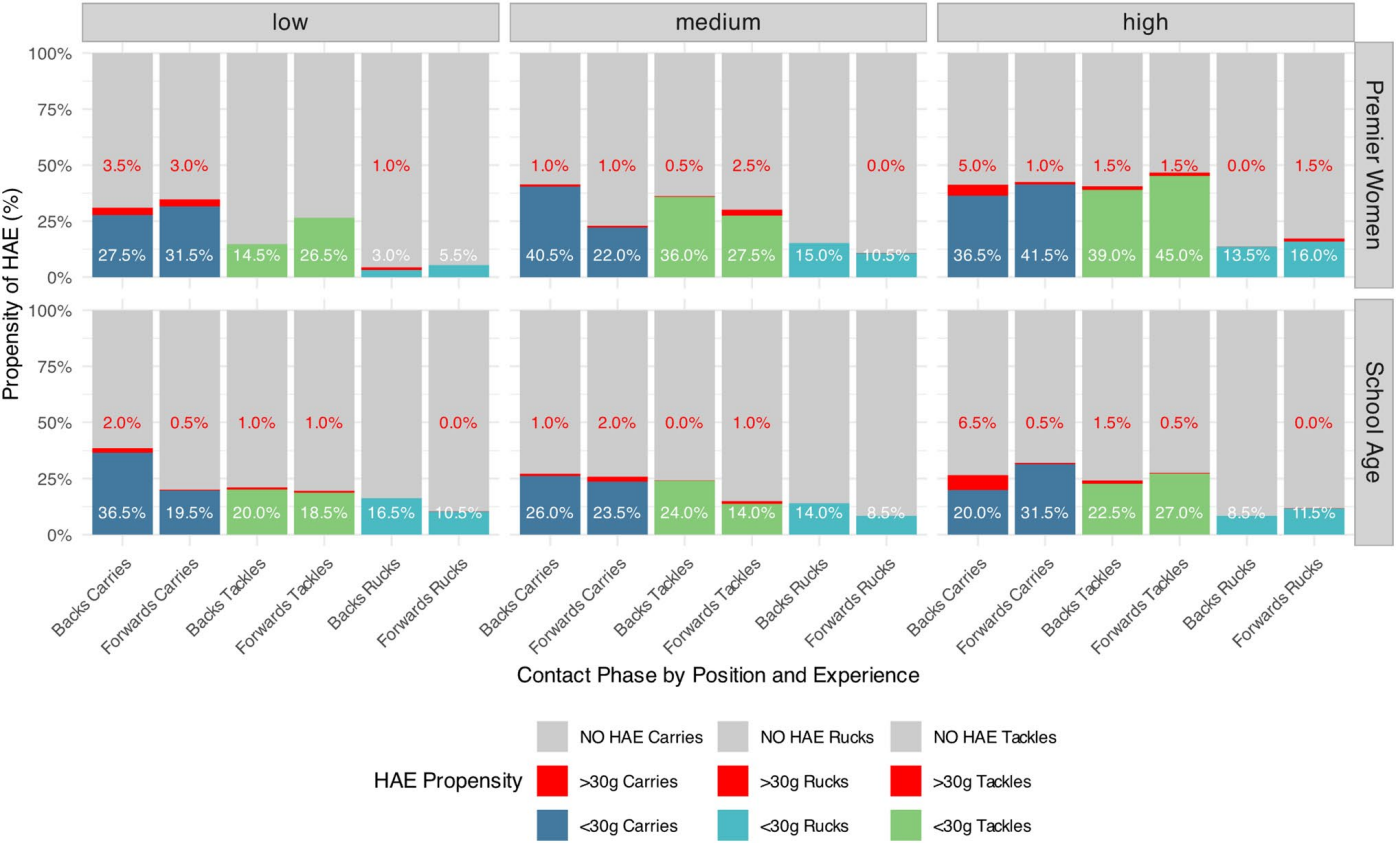
A bar chart displaying the propensity of head acceleration events (HAEs) by playing position (forwards, backs) and experience levels (low, medium, high) among female rugby players during contact phases (tackles, carries, and rucks) of a rugby match. The chart is divided into two main sections, Premier grade (top row) and school-age grade (bottom row). The red sections of the bar represent the propensity of > 30-g events

A total of 2917 (2421 direct or indirect HAE mechanisms, 496 unclear) video-verified HAEs were associated with the 8518 contact events described in Fig. 3. The focus for this analysis is tackles, ball carries, and rucks, thus scrums/lineouts/mauls were not further analysed in this study. Figure 4 shows the propensity for HAE during the contact phase. Premier high-experience forwards appear to have the highest propensity for tackles and carries compared with all other grades, levels, and positions. In the tackle (including both tackles and carries), Premier high-experience players had a significantly higher likelihood of experiencing an HAE compared with school-aged high-experience players (RR 1.21, 95% CI 1.06–1.37; *p* = 0.004). In those with medium experience, the Premier players showed a significantly greater likelihood of sustaining an HAE in both tackles (RR 1.78, 95% CI 1.41–2.25; *p* < 0.001) and carries (RR 1.65, 95% CI 1.21–2.26; *p* = 0.001) compared with the school-age grade. There was no significant difference in HAE propensity among low-experience players between the grades for either carries (RR 1.11, 95% CI 0.88–1.35; *p* = 0.33) or tackles (RR 0.78, 95% CI 0.49–1.01; *p* = 0.08). Regardless of grade or experience, the ball carriers bear the highest proportion of > 30-g events (Fig. 4).

#### HAE Propensity: Effect of Experience and Position Within Grade

To understand the effect of years of experience within the playing grade, comparisons were made between the low- and high-experience players by position, regardless of contact phase. The results present opposing trends between position and experience between Premier and school-age players. Premier high-experience forwards had a significantly higher likelihood of experiencing an HAE (RR 1.38, 95% CI 1.01–1.91; *p* = 0.04) compared with the low-experience forwards, while there was no significant difference in HAE likelihood between the high- and low-experience backs (RR 0.8, 95% CI 0.6–1.08; *p* = 0.16) (Fig. 4). In contrast, School-age high-experience backs had a significantly lower HAE likelihood (RR 0.78, 95% CI 0.64–0.94; *p* = 0.025) compared with low-experience backs (Fig. 4). There was no significant difference in HAE propensity between high- and low-experience forwards (RR 0.92, 95% CI 0.75–1.13; *p* = 0.466).

## Discussion

This study aimed to examine head acceleration exposure in a cohort of female rugby players who were representative of the range of age and skill levels in grass roots sport. The results of our study indicate that the risk of experiencing an HAE during the contact event in female rugby players is influenced by both playing grade and years of experience.

### Weekly Exposure

Premier women experienced the most frequent and severe weekly HAE exposure compared with all other female cohorts. This could be attributed to the higher intensity and physicality of play at the premier level, leading to a greater number of high-acceleration events. Compared with males, females experienced 1.6 times more low-magnitude HAEs (10–29 g), but only 0.6 times as many high-magnitude (> 30 g) events during match play [10]. However, the reverse seems to be true for training sessions. During trainings, females experienced 1.6 times more > 30-g events compared with males. Differences in exposure could stem from various factors, such as the nature/intensity of the training sessions, session structure, or the length of the pre-season. Minor discrepancies in data capture relative to the team’s season may have influenced training intensity, possibly influenced by pre-season groundwork. For instance, the male Premier pre-season is typically twice as long as their female counterparts, which may allow for more gradual physical conditioning and skill development. Bussey et al. suggested that the lower HAEs captured during the Premier men’s in-season training sessions may result from pre-season skill refinement, allowing players to focus more on team tactics and engage in less risky activities for HAEs during the early playing season [10]. Future research should examine the context of the > 30-g mechanisms during training sessions to determine the differences in the nature of the training environment and how they contribute to head acceleration exposure.

The amount of time spent exposed to rugby differed significantly (65–79%) between club and high school players, with club players having a notably higher total rugby exposure. One might expect this marked difference in exposure to have a significant impact on cumulative weekly HAE load. However, when looking at the youngest athletes (U13 and U15), there was no substantial difference in the HAE 10–29 g burden despite U13s having 65% more exposure time than U15s. However, we observed a 66% difference in > 30 g weekly IR associated with training, where the U15s far exceeded the U13s, despite the significantly lower exposure time. This suggests that factors other than total exposure time, such as skill level or physical development, may contribute to differences in HAE exposure. The training environment within the U15 age group may be marked by a multitude of challenges. Limited resources, such as coaching staff and equipment, combined with the wide range of rugby experience make it challenging for coaches to effectively match the training activity and players to the appropriate skill levels. In contrast, the clubs where the U13s play are generally well-established institutions, some of which have been around for more than a century. They have substantial memberships that support multiple playing grades and provide access to a wide range and depth of resources, including highly experienced coaching staff. Evidence from the literature suggests that participating in a higher-skilled and better-resourced rugby environment may reduce injury incidence in youth rugby [33]. The presumption is that settings with greater resources and skilled players offer a greater focus on strength and technique development, have a higher degree of athlete oversight, and may also benefit from more experienced coaching staff [33–35].

### Effect of Playing Experience on HAE Frequency and Magnitude

Rugby playing grades in New Zealand are primarily structured around the chronological playing age. Therefore, it is expected that the mean age of the U13 grade would be < 13 years, and the mean age of the U15 grade would be < 15 years. This was generally the case (as shown in Table 1 of Bussey et al., 2023), and was reflected by a strong correlation between playing grade and training age or years of rugby playing experience [10]. When comparing our male and female cohorts, it appears that male players have higher mean years of rugby experience than female players, and this trend is consistent with the U13 to Premier grades. Furthermore, the standard deviation is generally higher in the female data, indicating a wider spread of experience levels among female players within a playing grade. This is a common phenomenon in women’s rugby, as the majority of female players (70%) start playing rugby in their teenage years, with a large proportion picking up the sports in high school or university [1]. In comparison, the majority of male players began playing rugby between the ages of 4 and 12; only 26% reported a starting age between 13 and 17 years old [36]. Therefore, we examined the effect of years of rugby experience on HAE IR and magnitude in our female cohort instead of maintaining chronological age grades.

Comparing our current hourly HAE incidence findings with our previously published male cohort, we see similarities in the rate of accumulation of 10 to 29-g events, which accumulate at 4–8 times the rate of > 30-g events [10]. The most experienced Premier women exhibited the greatest number of HAEs across all thresholds for both the PLA and PAA. Their IR is similar to the elite female population reported by Tooby et al. (2023), which was not surprising as many of our senior Premier women players played in the FPC league, the highest grade of amateur women’s rugby in New Zealand [24]. However, our results further demonstrated a significant interaction between playing grade and years of experience in relation to the incidence of HAEs per hour of exposure. Specifically, we observed that the least-experienced school players demonstrated markedly higher incidence rates for events surpassing the 20-g and 2000-rad/s thresholds than their least-experienced Premier counterparts. This effect appears to be particularly pronounced for PAA compared with PLA, suggesting that younger players may experience heightened PAA during impact events. To fully understand these observations, a deeper analysis of HAE mechanisms is essential. Potential explanations could include differences in strength, physique, or neuromuscular control among younger players, as indicated by previous research [37–39]. Alternatively, there may be a significantly greater accumulation of inertial HAE mechanisms in the younger cohort, as these mechanisms typically entail higher rotational components than direct contact mechanisms [10].

Linear regression analysis further supports our suspicion regarding the effect of years of experience on PAA values. In school-age players, there is a significant inverse relationship between years of playing experience and the magnitude of rotational acceleration, which is much stronger than the relationship in Premier players. This would make sense if we expect that as players become more experienced, technique improves as does sport-specific strength and motor control, which may lead to an improvement in technique [40, 41]. In the Premier players, the relationship between PAA and experience is dependent upon the playing position, with backs experiencing higher PAA and forwards a lower PAA with increasing years of experience. The interaction was stronger for training sessions, which might reflect the differences in activities performed in unit drills. Forwards tend to spend more units time in scrums and lineouts, which may explain the lower incidence of HAE, while backs may spend more time doing tackles and rucks in open play. Forwards generally carry higher collision loads during match sessions [42] and as identified in our current cohort, more senior forwards carry a higher proportion of the contact load in matches.

Contradictory findings currently exist regarding the role of playing experience in sports-related concussion. For example, Swain et al. (2016) found that fewer years of rugby participation were associated with a higher injury incidence rate in amateur rugby, suggesting a potential relationship between playing experience and injury risk [43]. Yet, Quarrie et al. identified playing grade as a more significant risk factor over years of experience, with more injuries reported by higher-grade players [44]. The results of this study may provide a resolution to the conflicting literature, as they illustrate an increased PAA IR and magnitude in lower-experienced players competing in lower grades as well as in higher-experienced players competing in higher grades.

### Propensity for HAE During Match Contact Phases

In both the school-age and Premier cohorts, players with more experience participated in a higher proportion of carriers, rucks, and tackles. In the Premier women, the proportion of events increased consistently with player experience; the most experienced players appeared in approximately 50% of contact events, while the least experienced players accounted for less than 20% of contact events. A similar pattern was observed in the school-age cohort, where the high-experienced players, although forming the smallest sample proportion, accounted for the largest percentage of carriers and rucks. This trend demonstrates that more experienced players are have a higher level of involvement in contact events. There are two potential explanations for this finding. First, particularly within the Premier grade, the more experienced players are getting more playing time compared with the less experienced players, and more playing time would result in higher exposure to contact events. Second, it is likely that experienced players, particularly school-aged ones, are more confident in approaching contact situations and are therefore more likely to engage in them [45].

This study highlights the propensity for HAEs during contact phases, with a focus on the relative risk of experiencing an HAE based on grade and player experience. The findings reveal that Premier women have a higher likelihood of sustaining an HAE than schoolgirls. However, the effect of playing position and experience were markedly different. School-age low-experience backs have a higher likelihood of experiencing an HAE, while in the Premier grade it was the high-experience forwards with the highest HAE propensity. This finding is also in contrast to the male dataset [10], which identified the U19 forwards as having the highest HAE burden. For both the Premier and school-age groups, ball carriers have the greatest propensity for high-magnitude HAEs, regardless of experience. The latest findings indicate that female community players are potentially exposed to a risk of concussion injury when carrying the ball into contact, which is consistent with observations made among elite female players [6] as well as high-school players in Canada [46, 47].

### Limitations

This study was not without limitations. First, the quality of the iMG fit may be worse in smaller players as well as those with a narrow dental arch, triangular bite, and missing or misaligned teeth. However, we took several measures to address this issue, including having qualified dentists perform the iMG fitting and tracking the iMG-tooth displacement from the proximity sensor, which provided a fit quality score. Second, our cohort of U13 female players was small. Typically, in our region there are not enough U13 female rugby players to field an entire team or grade at this level. Therefore, younger females play in a mixed league run by local clubs, typically with only one or two females per team. On average, these females are one year older than their male teammates [10] because of the special dispensation offered to skilled female players who may otherwise not have teams to play on. Although we had nine U13 females in this cohort, we were only able to obtain data from seven of these individuals. Six of these players also played with U15 or U19 teams in 2021. Owing to the overlap in the school and club seasons, these players may have the opportunity to play more than one match per week, which would substantially affect their total exposure. However, none of our U13 cohort participated in more than one match per week during the study period. Third, our 2021 season was disrupted by a COVID-19 lockdown, which disrupted the U15 playing season. Therefore, we had to resume our tracking of this group in 2022. Such seasonal variations may affect the intensity of play or training, although the mixed-model procedure adopted in the analysis should accommodate these variations.

## Conclusions

This study sheds light on the complex interplay between playing grade, experience, position and head acceleration exposure (HAE) among female community rugby players. Premier women experience the highest frequency and severity of weekly head acceleration exposure compared with the younger female cohorts, reflecting the intense and physical nature of play at that level. However, exposure differences between club and high-school players revealed nuanced patterns, suggesting factors beyond total time exposure influenced HAE accumulation. Notably, the inverse relationship between playing experience and head acceleration magnitude underscores the importance of skill development and technique refinement over time.

These findings highlight the multifactorial nature of HAEs in female rugby and underscore the importance of considering factors such as playing grade, level of experience, and match contact phase when evaluating HAE and injury risk. Future research should explore the contextual factors influencing HAE mechanisms both in matches and trainings, informing the development of effective injury prevention measures.

## Supplementary Information

Below is the link to the electronic supplementary material.

Supplementary file1 (PDF 185 KB)

## Declarations

### Patient and Public Involvement

Patients and/or the public were not involved in the design, conduct, reporting, or dissemination of this research.

### Data Availability Statement

Data are available on reasonable request to the corresponding author.

### Code Availability

Custom codes for data processing are hosted on GitHub and can be shared upon request after all manuscripts have been accepted for publication.
