# Supplementary material for: Assessing Head Acceleration Events in Female Community Rugby Union Players: A Cohort Study Using Instrumented Mouthguards
Source: Sports Med. 2024 Sep 5;55(2):499–512. doi: 10.1007/s40279-024-02111-3 (PMC11946941; doi:10.1007/s40279-024-02111-3)
Supplement: Supplementary file 1 — Supplementary file1 (PDF 185 KB) [file 40279_2024_2111_MOESM1_ESM.pdf]

Supplementary Materials

Title: Assessing Head Acceleration Events in Female Community Rugby Union Players: A Cohort Study Using Instrumented Mouthguards

Melanie D Bussey[1], Danielle Salmon[2], Bridie Nanai[1], Janelle Romanchuk[1,2], Raul M Gomez[1], Darryl Tong, Gisela Sole, Ross Tucker [3,4], Éanna Falvey[4,5]
1 School of Physical Education, Sports and Exercise Sciences, University of Otago;2 New Zealand Rugby;3 Institute of Sport and Exercise Medicine, University of Stellenbosch; 4 World Rugby, Dublin, Ireland; 5 School of Medicine & Health, University College Cork, Ireland

Associate Professor Melanie Bussey (corresponding author) melanie.bussey@otago.ac.nz
Dr Danielle Salmon danielle.salmon@worldrugby.org
Bridie Nanai bridie.nanai@otago.ac.nz
Janelle Romanchuk janelle.romanchuk@postgrad.otago.ac.nz
Professor Darryl Tong darryl.tong@otago.ac.nz
Professor Gisela Sole gisela.sole@otago.ac.nz
Raul Martin Gomez marra610@student.otago.ac.nz
Professor Ross Tucker ross@sportsscientists.com
Professor Éanna Falvey Eanna.Falvey@worldrugby.org

Word count 4618; Tables 3; Figures 4; Supplementary Tables 1;

Grade	Experienc	HAE Threshold (g)	PAA (rads/s^2)							
			Match HAEs				Training HAEs			
			Count	Count %	Median	IQR	Count	Count %	Median	IQR
Premier Women	Low	<= 10.0	201	45.80%	537.8	-488.1	136	51.10%	532.9	-440.8
		10.1 - 20.0	159	36.20%	1178.6	-911.9	99	37.20%	1254	-821.4
		20.1 - 30.0	35	8.00%	2071.4	-1204.5	19	7.10%	2575.9	-1240.6
		30.1 - 40.0	17	3.90%	3605.2	-2110.1	9	3.40%	3421.1	-1046.1
		40.1 - 50.0	11	2.50%	5440.6	-1847.5	1	0.40%	4191	0
		50.1 - 60.0	8	1.80%	4136.7	-2679.1	0	0.00%	0	0
		60.1+	8	1.80%	5433.3	-4926	2	0.80%	5652.3	-3913.7
	Medium	<= 10.0	429	53.80%	456.8	-509.1	155	31.50%	531.7	-478.8
		10.1 - 20.0	263	33.00%	1062.5	-738.1	190	38.60%	1376.4	-1034.2
		20.1 - 30.0	65	8.10%	2323.4	-1096.2	85	17.30%	2417.5	-1017.1
		30.1 - 40.0	18	2.30%	3769.7	-2291	36	7.30%	3245.3	-1164.1
		40.1 - 50.0	11	1.40%	3947.4	-2468.6	13	2.60%	4664.6	-1219
		50.1 - 60.0	3	0.40%	6203.7	-6261	3	0.60%	5564.5	-1624.6
		60.1+	9	1.10%	8224.4	-5338.6	10	2.00%	7920.9	-2888.7
	High	<= 10.0	540	41.20%	624.8	-447.3	165	24.30%	539.2	-478.3
		10.1 - 20.0	540	41.20%	1131.1	-879.4	300	44.20%	1455.1	-917.6
		20.1 - 30.0	135	10.30%	2295	-1589.4	141	20.80%	2723.7	-910.2
		30.1 - 40.0	61	4.70%	3198.7	-2475.4	30	4.40%	3656.1	-788
		40.1 - 50.0	14	1.10%	3363.2	-1961.7	20	2.90%	5205.8	-1441.9
		50.1 - 60.0	12	0.90%	4394.8	-3453.2	11	1.60%	6215.7	-1529
		60.1+	8	0.60%	8268.6	-2144.7	12	1.80%	6763	-2350.4
School Age	Low	<= 10.0	428	33.50%	817.6	-584.6	296	28.50%	992.2	-704.8
		10.1 - 20.0	568	44.50%	1717.5	-972.1	506	48.70%	1816.5	-664
		20.1 - 30.0	200	15.70%	2840.1	-1216.2	159	15.30%	3054.3	-964.4
		30.1 - 40.0	44	3.40%	4060.4	-2294.9	53	5.10%	4494.7	-1250.8
		40.1 - 50.0	22	1.70%	5018.2	-1226.4	15	1.40%	5367.3	-1308.3
		50.1 - 60.0	12	0.90%	6470.3	-2630.7	5	0.50%	6315.9	-2244.3
		60.1+	2	0.20%	8436	-2343.2	4	0.40%	8442.4	-1688.7
	Medium	<= 10.0	125	43.90%	564.3	-478.7	73	44.00%	704.9	-779.5
		10.1 - 20.0	110	38.60%	1195.8	-981.1	62	37.30%	1629.7	-905.1
		20.1 - 30.0	27	9.50%	2198	-1789.2	18	10.80%	2583.3	-1398
		30.1 - 40.0	13	4.60%	3024.3	-2288.5	6	3.60%	3924.7	-2114.8
		40.1 - 50.0	4	1.40%	2576.1	-1614.4	2	1.20%	6990.2	-382.2
		50.1 - 60.0	2	0.70%	2991.7	-297.9	3	1.80%	5544.9	-346.5
		60.1+	4	1.40%	7286.4	-4077	2	1.20%	7633.2	0
	High	<= 10.0	177	45.70%	601	-408.4	91	63.20%	553.9	-450.1
		10.1 - 20.0	144	37.20%	1268.7	-1022.7	47	32.60%	1547.4	-1159.7
		20.1 - 30.0	35	9.00%	2090.3	-1301	5	3.50%	2370.3	-1104.6
		30.1 - 40.0	12	3.10%	2548.7	-1960.8	1	0.70%	3167.9	0
		40.1 - 50.0	10	2.60%	4018	-1704.1	0	0.00%	0	0
		50.1 - 60.0	4	1.00%	6171.3	-1662.7	0	0.00%	0	0
		60.1+	5	1.30%	7411.8	-3108.8	0	0.00%	0	0
